# COVID-19 Pathophysiology Predicts That Ischemic Stroke Occurrence Is an Expectation, Not an Exception—A Systematic Review

**DOI:** 10.3389/fneur.2020.607221

**Published:** 2021-01-28

**Authors:** Tissa Wijeratne, Sheila Gillard Crewther, Carmela Sales, Leila Karimi

**Affiliations:** ^1^School of Psychology and Public Health, La Trobe University, Melbourne, VIC, Australia; ^2^Department of Neurology, Western Health and University Melbourne, Australian Institute of Muscular Skeletal Sciences (AIMSS), Level Three, Western Health Centre for Research and Education (WHCRE), Sunshine Hospital, Melbourne, VIC, Australia; ^3^Department of Medicine, Faculty of Medicine, University of Rajarata, Anuradhapura, Sri Lanka; ^4^Faculty of Social and Political Sciences, Ivane Javakhishvili Tbilisi State University, Tbilisi, Georgia

**Keywords:** COVID-19, stroke, ACE2, cytokines, pathogen associated molecular pattern, post Covid19 neurology syndrome, PCNS

## Abstract

Clinical reports of neurological manifestations associated with severe coronavirus disease 2019 (COVID-19), such as acute ischemic stroke (AIS), encephalopathy, seizures, headaches, acute necrotizing encephalitis, cerebral microbleeds, posterior reversible leukoencephalopathy syndrome, hemophagocytic lymphohistiocytosis, peripheral neuropathy, cranial nerve palsies, transverse myelitis, and demyelinating disorders, are increasing rapidly. However, there are comparatively few studies investigating the potential impact of immunological responses secondary to hypoxia, oxidative stress, and excessive platelet-induced aggregation on the brain. This scoping review has focused on the pathophysiological mechanisms associated with peripheral and consequential neural (central) inflammation leading to COVID-19-related ischemic strokes. It also highlights the common biological processes shared between AIS and COVID-19 infection and the importance of the recognition that severe respiratory dysfunction and neurological impairments associated with COVID and chronic inflammation [post-COVID-19 neurological syndrome (PCNS)] may significantly impact recovery and ability to benefit from neurorehabilitation. This study provides a comprehensive review of the pathobiology of COVID-19 and ischemic stroke. It also affirms that the immunological contribution to the pathophysiology of COVID-19 is predictive of the neurological sequelae particularly ischemic stroke, which makes it the expectation rather than the exception. This work is of fundamental significance to the neurorehabilitation community given the increasing number of COVID-related ischemic strokes, the current limited knowledge regarding the risk of reinfection, and recent reports of a PCNS. It further highlights the need for global collaboration and research into new pathobiology-based neurorehabilitation treatment strategies and more integrated evidence-based care.

## Introduction

Since the first case of coronavirus disease 2019 (COVID-19) infection was reported in Wuhan, China in December 2019, a significant number of thrombotic complications affecting the venous and arterial systems have been published in the literature (1–3), with the World Stroke Organization now recognizing that acute ischemic stroke (AIS) increases the severity of severe acute respiratory syndrome coronavirus 2 (SARS-CoV2) viral infection by 2.5-fold (4, 5). Similar associations between systemic infections, inflammation, and AIS are longstanding (6). However, to date, few reports are reviewing the molecular bases of the peripheral and central mechanisms induced by SARS-CoV2 infection and potential neurological manifestations with focused attention on AIS.

Neurological manifestations of COVID-19 infection were first described by Mao et al. who observed six cases of acute cerebrovascular disease (2). Subsequently, 19 cases of COVID-19-related strokes particularly affecting the young and involving medium to large arteries were reported from a tertiary center in New York (3), highlighting the need for better understanding of the potential mechanisms linking COVID-19 and AIS.

Stroke has been associated with other earlier coronavirus infections. In 2002, AIS was first detailed by Umapathi et al., in 4 of 206 patients who presented with large vessel occlusion associated with SARS-CoV infection in Singapore in 2002 (7). Two other patients with AIS were also described in Middle East respiratory syndrome corona virus (MERS-CoV)-infected patients in Saudi Arabia during the 2015 epidemic (8).

SARS-CoV2 is known to initially bind to the angiotensin-converting enzyme 2 (ACE2) receptors of epithelial and endothelial cells where an immediate immunological activation occurs that can, in severe cases, eventually lead to hypercoagulability or thrombophilia and increased tendency of clots forming in the blood and potentially AIS (9). However, ŧhere is limited information on the physiological abnormalities and mechanisms linking COVID-19 and AIS, although a number of mechanisms have been proposed. The major mechanisms that have been proposed to date include systemic innate immunity-mediated hyperinflammation, neurovascular endothelial dysfunction, endotheliitis, central nervous system renin–angiotensin–aldosterone system (RAAS) dysregulation, oxidative stress, and excessive platelet aggregation (10–12). Thus, this scoping review aimed to elucidate potential pathophysiological mechanisms predisposing patients with COVID-19 to a higher risk for neurovascular events.

## Methodology

The authors of this scoping review used the Arksey and O'Malley methodology to identify and extract useful literature (13). The steps undertaken include (1) research questions identification; (2) relevant literature identification; (3) screening and selection of relevant literature; (4) data charting; and (5) analyzing, summarizing, and reporting the results.

### Identification and Development of the Research Question

This focused on the general research question: “Are there mechanisms associated with COVID-19 infection that are likely to predispose patients to ischemic stroke?”

The following are the specific areas of interest:

a. Can current understanding of the immunological mechanisms associated with the inflammatory responses to severe COVID-19 potentially predispose a patient to AIS?

b. Are there other potential pathophysiological mechanisms predisposing COVID-19 patients to upregulate procoagulable mechanisms leading to thrombi formation and potentially AIS?

### Relevant Literature Identification

A literature review of articles regarding cases and mechanisms underlying the co-occurrence of COVID-19-and AIS, published in English between January 2000 and August 12th, was carried out up to August 15th, 2020. Only studies (qualitative and quantitative studies, systematic reviews, metanalysis, case reports, and case series) that directly or indirectly link pathophysiological mechanisms to ischemic stroke and COVID-19 infection have been included in the final review (see Table 1 detailing the studies).

**Table 1:** Summary of the main studies on the mechanisms of COVID-19-related strokes.

**Publication date**	**Author**	**Type of study**	**Objectives**	**Conclusion**
July 18, 2020	Kempuraj et al. (14)	Review article	To understand the relationship between COVID-19 infection and the neuroinflammatory responses in the brain, especially concerning psychological stress, mast cell activation, and cytokine storm–associated responses.	COVID-19 19 can worsen neuroinflammation thru activation of mast cells, neurons, astrocytes, microglia, endothelial cells, and increase inflammatory cytokine and chemokine levels in the CNS
June 26, 2020	van den Berg et al. (15)	Review article	To review the role of NLRP3 inflammasome dysregulation in the severe COVID-1919 infection.	NLRP3 inflammasome and its associated pathways plays an important role in severe COVID-19 19 infection especially in patients with impaired immune function and predisposes patients with pre-existing suboptimal immune responses to poor outcomes.
June 19, 2020	Rodrigues-Diez et al. (16)	Review article	To discuss the potential benefits of statins in COVID-1919 infection.	Statins could reduce viral replication by autophagy activation and exert anti-inflammatory properties particularly on NF-κB and NLRP3 inflammasomes. It can also modulate coagulation responses.
April 14, 2020	Zuo et al. (17)	Prospective Cohort study	To assess the role of neutrophil extracellular traps (NETs) in COVID-19 19 infection and identify the relationship to severity of illness.	High levels of NETS in patients with COVID-19 19 may contribute to cytokine release and respiratory failure.
June 2, 2020	Tomar et al. (17)	Review article	To identify the role of enhanced neutrophil infiltration and the release of NETs, complement activation and vascular thrombosis during necroinflammation in COVID-19	NET formation induces production of proinflammatory cytokines leading to tissue inflammation responsible for cytokine storm and sepsis.
June 29, 2020	Middleton et al. (18)	Prospective Cohort study	To assess the role of neutrophil extracellular traps (NETs) in COVID-19 19 infection and identify the relationship to severity and progression of illness.	Neutrophils of COVID-19 19 patients displayed excessive NET at baseline and was blocked by neonatal NET-Inhibitory factor. Levels also correlate with intubation and death.
May 2010	Hermus et al. (19)	Review article	To identify the biomarkers associated with carotid plaque formation	Various biomarkers linked to inflammation, lipid accumulation, thrombosis and angiogenesis have been related to plaque formation and vulnerability.
July 2020	Mohamud et al. (20)	Case series	To describe six cases of COVID-19 positive patients with associated intraluminal carotid artery thrombus.	Inflammation related to COVID-19 19 may lead to plaque rupture of previously known vulnerable atheroma leading to thrombosis an ischemic stroke.
June 27, 2020	Ueland et al. (21)	Letter to the editor/prospective Cohort study	To identify relationship of plasma markers reflecting inflammation and fibrosis and respiratory failure in hospitalized COVID-19 patients.	Methyl-metalloproteinases (MMP) may be an early indicator of respiratory failure in COVID-19 patients and underscore the role ECM remodeling and fibrosis in this disorder.
June 5, 2020	Solun et al. (22)	Review article	To review the role of matrix MMP and the kinin-kallikrein system (KKS) in the pathomechanism associated with COVID-19 19 related acute lung injury.	Overexpression of MMP results in acute lung injury and remodeling among COVID-19 19 patients and the use of its inhibitor may be a potential therapeutic strategy.
June 29, 2020	Schönrich et al. (23)	Review article	To review the role of overproduction of reactive oxygen species (ROS) in local and systemic tissue damage associated with COVID-19 19 infection.	ROS increases the formation of NETs and suppresses the adaptive immune system.
May 15, 2020	Wright et al. (24)	Prospective Cohort	To determine the correlation between thromboelastography measurements of coagulation and thromboembolic events in COVID-19 19 patients.	Failure of clot lysis at 30 min on thromboelastography is predictive of thromboembolic events in critically ill COVID-19 19 patients.
July 17, 2020	Kunutsor et al. (25)	Meta-analysis	To identify the association of CRP and VTE risk.	Elevated CRP is associated with greater VTE risk, consistent with a linear dose–response relationship.
July 9, 2020	Zhang et al. (26)	Cross-sectional study	To determine the coagulation profiles of routine hemostasis tests, natural anticoagulants, coagulant factors and antiphospholipid antibodies in critically ill COVID-19 patients.	The low activities of natural anticoagulants, elevated factor VIII level and the presence of antiphospholipid antibodies, together, may contribute to the etiopathology of coagulopathy in COVID-19 patients.
June 11, 2020	DiNicolantonio et al. (27)	Review article	To identify the role of endothelial tissue expression of tissue factor in COVID-19 infection	SARS-CoV-2 infection of endothelial cells evokes the expression of TF which is contingent on endosomal NADPH oxidase activation.
June 9, 2002	Bautista-Vargas et al. (28)	Review article	To identify the role of tissue factor (TF) in the hypercoagulability associated with COVID-19 19 infection.	TF may be a critical mediator associated with the development of thrombotic phenomena in COVID-19.
September 2015	Saha et al. (29)	Review article	To identify the mechanisms associated with tissue factor production and atherothrombosis.	TF is essential in the initiation of the extrinsic pathway of the coagulation cascade and appears to be a critical determinant of atherosclerotic plaque thrombogenicity.
December 5, 2002	Akerström et al. (30)	Experimental study	To identify the role of nitric oxide in SARS-CoV infection.	SARS-CoV infection inhibits viral replication by affecting RNA replication and reduction in expressed spiked protein production.
July 2020	Cheng et al (31)	Review article	To identify the correlation between angiotensin-converting enzyme 2 (ACE2) and severe risk factors for coronavirus disease 2019	ACE2 is an essential part of the RAS, and it has extensive vascular and organ protection functions in hypertension, diabetes, cardiovascular disease, and ARDS
May 7, 2020	Hess et al. (32)	Review article	To identify the role of ACE in COVID-19 related strokes.	Binding to and depletion of ACE2 may tip the RAS balance in favor of the ACE-1-angiotensin II-AT1 axis and contribute to endothelial dysfunction, organ damage, and stroke.
August 22, 2008	McColl et al. (6)	Review article	To explore the impact of systemic infections and inflammation on stroke susceptibility	The role of systemic infections and inflammation in stroke (onset of the stroke as well as post stroke outcome)
July 2, 2020	Ellul et al. (33)	Review article	To identify the clinical manifestation of neurological disorders caused by COVID-19	The wide assortment of neurological disorders affecting 901 patients by May 2020 (at the time of the review)
August 7, 2019	Albensi B. C. (34)	Review article	To explore the importance and science around NF-?B activity with a view to correlate this with COVID-19 and immune involvement	NF-?B is an important transcription factor with critical roles in mitochondrial function, apoptosis, mechanisms of disease in COVID-19 and brain involvement as well
September 3, 2020	Wijeratne et al. (35)	Review article	To identify the role of inflammation affecting vascular systems as well as the importance of NLR as a potential biomarker in this context	NLR is a useful and easily available biomarker in atheromatous vascular disease supporting the same role in COVID-19 and large vessel disease
June 2020	Boldrini et al. (36)	Special article	To identify the impact of COVId-19 outbreak on rehabilitation services in Italy	Increasing pressure from the acute services to transfer the patients to rehabilitation services while the challenges to provide rehabilitation services in the outpatient as well as home settings due the restrictions
June 25, 2020	Varatharaj et al. (37)	Research paper (nationwide, cross speciality surveillance study)	To identify the epidemiology of acute neurological and psychiatric complications of COVID-19 as reported by the national registry in UK.	One hundred and Twenty five patients with complete data set noted 57 had an ischemic stroke (74%), nine had intracerebral hemorrhage (12%) and one with (1%) CNS vasculitis. Thirty nine patients (31%) had altered mental status while 69% had a neuropsychiatric syndrome.
July 20, 2020	Spence et al. (11)	Review	To identify the mechanisms of stroke in COVID-19	Vasculitis, hypercoagulability, endothelialinjury, microvascular thrombosis, cytokine storm, systemic hypoxia, fresh DVT, cardiac alterations
September 10, 2020	Wijeratne et al. (38)	Case Report and review	1.To correlate the CRP, D-dimer levels, NLR ratio changes with variety of clinical events at the intensive care unit and neurorehabilitation ward. 2. To explore the utility of bedside tablet use such as MRF visual fields and Lee-Rayan eye-hand coordination test during neurorehabilitation	The in-depth case study illustrates the significant of close monitoring of simple blood tests such as CRP, NLR with predicable ability of clinical worsening through fast recognition of pattern changes.
June 26, 2020	South et al. (39)	Review	To identify the risk of stroke in the context of preceding infection, including COVID-19	SARS-CoV-2 global pandemic is not the first viral infection to be linked with stroke. The current pandemic is an ideal opportunity to acquire valuable insight toward the relationship between stroke and infections
September 2020	Sivan et al. (40)	Short communication (research paper)	To explore the authors, work on development of an integrated rehabilitation pathway with systematic, efficient tele-rehabilitation model	COVID-19 Yorkshire Rehabilitation Screen(C19-YRS) (previously developed telephone screening tool) can be incorporated toward an efficient, systematic, telerehabilitation pathway

### Screening and Selection of Relevant Literature

In the first step, MEDLINE, Cochrane, and Cumulative Index to Nursing and Allied Health Literature (CINAHL) databases were searched to identify useful keywords. Subsequently, the identified keywords were used to search the same databases for relevant studies. The literature was first screened at the title and the abstract level; then, full-text articles were assessed for eligibility. The manual bibliographic search of identified studies was also done in the last step of the literature search. The following keywords were used: COVID-19, coronavirus, mechanism, inflammation, thrombosis, embolism, endotheliitis, arteritis, neuroinflammation, and ACE2 receptors. Two researchers (CS and TW) reviewed relevant articles independently. Any disagreement for inclusion was resolved by a third author (LK).

### Data Charting and Analysis

The included studies were charted, and the following parameters were taken into account: publication year, type of study, aims and objectives of the study, and study findings and conclusion. Included studies were analyzed extensively and are listed in Table 1.

## Results and Analysis

**Figure 1:**
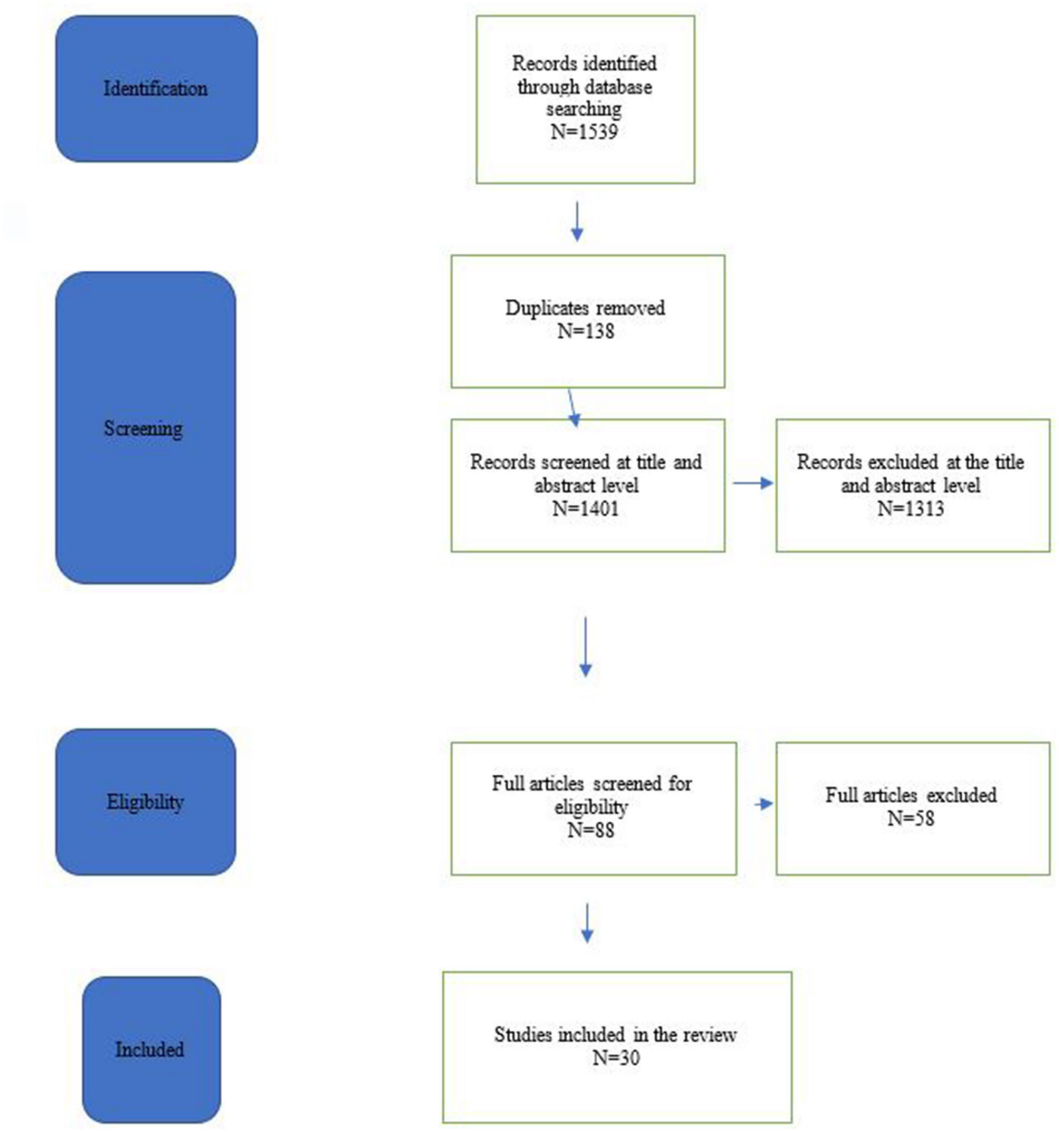
Prisma chart.

A total of 1,539 studies were identified in the electronic search. After removal of duplicates and screening at the title level, 230 articles were further reviewed at the abstract level, yielding 88 articles requiring assessment. Fifty-eight further articles were excluded, as these papers were not addressing the predefined research questions. A thorough review by two authors (CS and TW) deemed these papers to contain secondary information with repetition rather than an original contribution to the research questions. A total of 30 articles were included in the study. The details of the said studies are outlined in Table 1. The search process is summarized in a Preferred Reporting Items for Systematic Reviews and Meta-analyses (PRISMA) in Figure 1.

At the time of this submission (as of September 5th, 2020, WHO situation report), the global number of confirmed SARS-CoV2 cases were 26,171,112 with 865,154 confirmed deaths with a mortality rate of ~3.3%. It is also important to note that only a small proportion of COVID-19-infected individuals progress to severe disease, and of these, a smaller number experience stroke and/or death. The most likely predisposing factors to serious COVID-19 disease states are age, sex, and immune system inability to deal with environmental infection together with genetic factors, and associated cardiovascular risk comorbidities such as hypertension, diabetes, obesity, neurological disorders, and medication interactions. Indeed, it appears that exaggerated immune responses to infection and chronic inflammation may be the key factor in the pathogenesis of severe COVID-19 and associated cerebrovascular complications.

### Inflammatory Mechanisms of COVID-19

#### Initial Inflammatory Responses (Key References Are in Table 1; at the End of the Paper)

It is currently accepted that the SARS-CoV2 virus, like other coronaviruses, attacks host cells by binding to ACE2 (12, 41–43). Initial virus recognition occurs via the epithelial cells of the olfactory and respiratory tract of the infected person (39, 43) and would be expected to immediately activate the innate immune system of individual cells and the associated vascular supply (44). The cells within the respiratory epithelia are assumed to release the first set of cytokines such as tumor necrosis factor (TNF) and interleukin-6 (IL-6) (45) that then activate further proinflammatory macrophages and monocytes to accumulate in the alveoli.

The recruited monocytes and macrophages are a further rich source of cytokines and monocyte chemoattractant protein-1 with an additional army of immune-related cells coming to play a major component (45), as described in Tables 2, 3. At this stage, the majority of individuals will be asymptomatic, while some may experience flu-like symptoms, sore throat, myalgia, diarrhea, fever and headache, etc.

**Table 2:** Pathophysiology of COVID-19 and acute ischemic stroke.

**Pathophysiology of COVID-19 and Acute Ischemic Stroke—Chronological Order**
1. Binding of SARS-CoV-2 to ACE-2 on the surface of alveolar pneumocytes (ACE2 is the receptor for host cell entry of SARS-CoV-2. ACE-2 is widely expressed on the alveolar epithelial cells, endothelial cells, enterocytes of the small intestine and arterial smooth muscle cells) (46)
2. Viral infection activates intracellular pattern recognition receptors (PRRs) in the host. PRRs sense pathogen associated molecular patterns (PAMPs) [not this is very similar to pathophysiology of AIS where acute ischemia induced damage associated molecular patterns (DAMPs) perform exactly the same task] (10, 47–49)
3. PAMPs sets off cytolytic immune responses with type one interferon and natural killer cells (NK) with aim of removing the pathogens (50)
4. Endothelial activation secondary to direct viral activation
5. Innate immune response with the recruitment of macrophages, neutrophils, and monocytes to the alveoli.
6. Further innate immune reaction with T helper cells 1&17 T cells and ongoing recruitment of monocytes, macrophages and neutrophils (51)
7. Endothelial activation secondary to direct viral action
8. Endothelial activation secondary to persistent inflammation
9. Activation of cell adhesion molecules and further recruitment of T cells, monocytes and macrophages
10. Release of tissues factors from the activated endothelium
11. Recruitment of intravascular neutrophils
12. Release of neutrophil extracellular traps (NET) from the activated endothelium
13. Release of von Willebrand factor (vWF) and micro thrombosis (52)
14. Maladapted innate immune response and procoagulant activity lead to systemic cytokine rise
15. Increased platelet aggregation and thrombo-embolism
16. Activation of cell adhesion molecules and ongoing recruitment of monocytes, macrophages and neutrophils within the cerebral blood vessels
17. Endothelial activation in cerebral blood vessels
18. Endothelial dysfunction in the brain
19. Local immune and inflammatory activity in the brain
20. Disruption of the blood-brain barrier
21. Activation of microglia in the brain
22. Further recruitment of resident immune cells in the brain
23. Increased inflammatory activity in the brain
24. Micro-thrombosis, thrombo-embolism in the brain
25. Hypoperfusion of cerebral tissues
26. Acute Ischemic stroke

* *Attempts have been made to assimilate all available current evidence to formulate this table. However, we are aware the pandemic is still evolving, and additional molecular mechanisms and pathways are likely to be discovered as time goes by*.

**Table 3:** Summary of Pathophysiological mechanisms of COVDI-19 related Acute Ischemic Strokes.

Inflammatory mediators of ischemic stroke	The role of cytokines	• First released by the respiratory epithelium (45) • Further released by T lymphocytes activates other cellular mediators mediating secondary hemophagocytic lymphohistiocytosis (58, 59)
	NLRP3 inflammasomes	• Activated by viroporin protein 3a (63–65) • Drives production of inflammatory cytokines, pathogen-associated molecular patterns (PAMPs) and damage-associated molecular patterns (DAMPs) (63) • May induce plaque instability by overdriving response of cellular mediators such as macrophages, neutrophils, lymphocytes and vascular smooth muscle cell (70–72)
	Inflammation-induced plaque vulnerability	• Predominance of T cells, macrophages and neutrophils populating an atheromatous plaque leading to plaque rupture (73) • Characterized by elevation of proteolyic biomarkers such as metalloproteinases (MMPs) and cathepsin cysteine proteases (CCP's) (19, 21, 22, 75)
	Oxidized Low-Density Lipoproteins (oxLDL)	• A marker of increased oxidative stress (23) • COVID-19-related disruption of the receptor-mediated uptake of oxLDL (78)
	Neutrophil extracellular traps (NETs)	• Networks of chromatin, proteins and oxidant enzymes that protrude from membranes of activated neutrophil and mediate infection containment (17) • Also a marker of inflammation-related thrombosis (17, 25, 35, 101–106)
Coagulatory disfunction	D-dimer	• Mediates immunologic defense systems resulting in thrombi formation (107) • Increased level is a biomarker of fibrinolytic shutdown leading to massive fibrin formation resulting in thrombosis (108).
	Natural anticoagulants and antiphospholipid antibodies	• COVID-19 induced decrease in the amounts of physiological anticoagulants and increased levels of coagulant factors and antiphospholipid antibodies (26, 109, 110)
Endotheliopathy	Endothelium-driven activation of the extrinsic coagulation system	• Imbalance in the ACE2 and angiotension II (AT-II) receptors leads to the upregulation of tissue factor (27, 28) • Tissue factor interacts with Factor VII to activate the extrinsic coagulation system (27, 28)
	Endothelium-driven nitrous oxide deficiency	• COVI-19 related endotheliopathy results in the suppression of nitric oxide synthase (NOS), resulting in nitric oxide deficiency (30) • Results in loss of vasodilatory effect and promotes adhesion of platelets and leukocytes to the vessel wall (111)
Renin Angiotensin Aldosterone System (RAAS) and ACE-2 deficiency	Alterations in the balance between the classical RAAS and the ACE2 pathways	• Promotes organ damaging effects of the classical RAAS pathway (112) • Promotes overactivity of the sympathetic nervous system resulting in the exacerbation of traditional stroke risk factors (113)

The intrinsic pathogenicity of severe coronavirus infection in vulnerable people ensures that the acute severe inflammatory response to the COVID-induced respiratory distress results in decreased levels of circulating lymphocytes, secondary hemophagocytic lymphohistiocytosis (HLH) (46–50) shifts immune defenses toward natural killer (NK) and circulating macrophages (macrophage activation syndrome), and increased neutrophils. An early and important mediator to this phenomenon is the significant elevation of proinflammatory cytokines that fuel various processes in cerebrovascular ischemia (Tables 2, 3). Indeed, sepsis-induced stimulation of the immune system leads to a clonal expansion of antigen-specific T lymphocytes, which result in the further release of proinflammatory cytokines and activation of cells such as macrophages and cytotoxic T lymphocytes, which are otherwise known as the HLH (51, 52), and are thought to be caused by a dysfunction of the normal regulatory mechanisms (52). As a result of the build-up of proinflammatory immune responses, an imbalance favoring the mass increase in neutrophil to leukocyte ratio, and inflammatory cytokines such as TNF, interferon-γ, IL-1, IL-6, IL-18, and IL-33 propels global inflammatory activity (52–55), which is known to lead to multiorgan dysfunction among critically ill patients (45).

Nod-like receptor family, pyrin domain-containing 3 (NLRP3) inflammasomes play an important role in innate immunity and are directly activated by the virus itself via the small viral protein, viroporin protein 3a, that is known to modify cellular membranes and facilitate virus release from host-infected cells (56–58). This multiprotein complex in the cytosol drives a cascade of reactions resulting in the formation of proinflammatory cytokines such as interleukin-1β (IL-1β), interleukin-18 (IL-18), pathogen-associated molecular patterns (PAMPs), and damage-associated molecular patterns (DAMPs), which are usually tightly regulated in patients with normal immune systems (56). Animal studies related to SARS-CoV infection have demonstrated that the overactivation of the NLRP3 inflammasome contributes to the significantly increased virulence and the high incidence of acute lung injury (59, 60). This is also one of the rationales behind the Greek Study in the Effects of Colchicine in COVID-19 Complications Prevention (GRECCO) trial, which is investigating the utility of colchicine, as a non-specific inhibitor of the NLRP3 inflammasome, in the treatment of COVID-19 infection (61, 62). By inhibiting this pathway along with nuclear factor kappa B (NF-KB) inhibition of the innate immune response (63), statins have also been shown to be potentially beneficial for patients with COVID19 infection via intervention through this pathway (64).

In patients with stroke, it has been shown that the NLRP3 inflammasome plays a significant role in cerebral atherogenesis by similar activation of the immune system and increase in macrophages, neutrophils, lymphocytes, and vascular smooth muscle cell, which also play an important role in plaque instability (65–67). The neuronal cell death, which can be attributed to the NLRP3 inflammasome activation, has also been shown to be reversible with immunoglobulin in 3-month-old mice stroke models (65). However, in patients with impaired immune responses, such as the elderly, there is an unprecedented activation of the NLRP3 inflammasome coupled with mitochondrial dysfunction and increased proportions of the mitochondrial reactive oxygen species, which further worsen tissue damage and initiate cell death (66). In patients with obesity and diabetes in which NLRP3 inflammasomes are already basally activated, and the immune responses are suboptimal, viral-induced activation of the former may worsen a preexisting chronic inflammatory state (67). Hence, this factor may be related to the poor outcomes of elderly patients with COVID-19 and stroke and with traditional cardiovascular risk factors such as obesity and diabetes (10).

#### Inflammation-Induced Plaque Progression and Vulnerability

COVID-19 inflammation induces a prothrombotic milieu among patients who are at risk of vascular events and those with the pre-existing atheromatous disease. Among patients with coronary disease, evidence suggests that virally induced inflammatory infiltrates such as T cells, macrophages, and neutrophils populate the atheromatous plaque leading to a cascade of events including vascular permeability, endothelial disruption, and exposure of prothrombotic elements such as collagen, tissue factor, and platelet adhesion molecules, which all play a role in thrombogenesis (68). Furthermore, it is known that carotid artery plaques with features of a thin fibrous cap, large core lipid, intraplaque bleeding, and the abundance of monocyte-derived macrophages and activated smooth muscle cells cause instability and vulnerability to plaque rupture (69). The presence of proteolytic enzymes such as metalloproteinases (MMPs) and cathepsin cysteine proteases (CCPs), which are secreted by activated macrophages, promote degradation of the extracellular matrix and plaque fragility and potentially result in thromboembolism (16, 34) while making acute ischemic stroke a likely event in the potential trajectories of patients with systemic COVID-19 infection.

Indeed Mohamud et al. have described five cases of acute ischemic stroke associated with an intraluminal carotid artery thrombus with concomitant COVID-19 symptoms 0–14 days before the onset of stroke (70). A proposed mechanism of this co-occurrence is inflammation-related plaque rupture as manifested by the elevated levels of inflammatory biomarkers such as lactate dehydrogenase, C-reactive protein, ferritin, D-dimer, and interleukin (70). Another study reported a case of symptomatic intracranial stenosis in a confirmed COVID-19 patient in a hyperinflammatory state, as evidenced by elevated acute inflammatory biomarkers (71). A similar hyperimmune trend was described in another patient with symptomatic posterior circulation stenosis and concomitant stroke (72). While these biomarkers are not specific for plaque rupture, there is evidence to suggest tha tplaque-rupture-specific MMPs implicate temporal association with the onset of acute respiratory failure among COVID-19 patients (73). Moreover, it has been proposed that aprotinin, a protease inhibitor that inhibits MMPs, may provide benefit for patients with COVID-19-related acute respiratory distress syndrome in experimental studies (74). However, at least in severe cases, the use of aprotinin to inhibit clot breakdown may need to be cautioned if the likelihood of COVID-19 inducing a proinflammatory hypercoagulation response to the SARS-CoV2 virus is considered.

Another putative mechanism that can lead to plaque progression in patients with COVID-19 infection is the disruption of the receptor-mediated uptake of oxidized low-density lipoproteins (oxLDL) by the monocyte-derived macrophages with exposure to proinflammatory stimuli (19). Indeed, animal and human studies indicate that increased expression of lectin-like oxidized lipoprotein 1 receptor (LOX-1) is a risk factor for stroke and promotes restenosis among patients with preexisting cardiovascular disease (75). It is also evident that COVID-19 infection results in a disproportionate increase in reactive oxygen species, which translate to lipid oxidation and increased oxLDL levels (20). Thus, this is presumably another one of the reasons why elderly patients with preexisting cardiovascular risk have a higher propensity of developing severe COVID-19 illness (76) and AIS.

#### The Role of Peripheral Biomarkers to COVID-19 That Are Also Characteristic of Ischemic Stroke

The pathognomonic features of sepsis-induced inflammation among patients with coronavirus infection is manifested peripherally as neutrophilia in combination with marked lymphopenia (77). This is characterized by the marked increase in various inflammatory biomarkers such as the neutrophil to lymphocyte ratio (NLR), C-reactive protein, and serum ferritin (21–23, 78–84). In a prospective cohort study involving patients with concomitant COVID-19 and ischemic stroke, 9 of the 10 patients have elevated NLR (85). Similarly, the majority of AIS patients presented recently (86, 87) and in various COVID-19 and AIS case series has reported increased NLR values (72, 88–91). A significant degree of lymphopenia coupled with viral-induced neutrophilia and the migration of the neutrophils to the ischemic core may explain the elevated NLR among patients with ischemic stroke (92).

CRP is another inflammatory biomarker that is disproportionately increased among patients with coronavirus infection and AIS. The largest cohort study involving 32 ischemic stroke patients with concomitant COVID-19 infection reported that elevated CRP occurred in more than 90% of the patients (93, 94). All of the six ischemic stroke patients reported by Beyroutti et al. and three of the four patients observed by Tunc et al. showed elevated CRP levels (72, 89). AIS patients described in various case reports and case series with COVID-19 infection have also been reported to have raised CRP levels (16, 38, 91, 95–98) While CRP is a marker of inflammation, a meta-analysis likewise confirms its role in thromboembolic events (98). On the other hand, serum ferritin, an acute phase reactant and an inflammatory biomarker has also been reported to be elevated among patients with COVID-19 and AIS (85, 88, 89, 99, 100). Apart from its role in systemic inflammation, serum ferritin likewise predicts the degree of neural damage among patients with AIS as characterized by its correlation with markers of blood–brain barrier damage such as glutamate, interleukin-6, matrix metalloproteinase-9, and cellular fibronectin (35, 82).

#### Neutrophil Increase, Lymphocyte Decrease, and Neutrophil Extracellular Traps in Thrombin in General and Previously Associated With Acute Ischemic Stroke

As alluded to above, COVID-19 infection is associated with reduced leukocyte number and increased neutrophil to leukocyte ratio and so makes the recent evidence of dysregulated neutrophil extracellular traps (NETs) predictable (101–103). NETs are neutrophil-produced extracellular networks of chromatin, proteins, and oxidant enzymes that protrude from membranes of activated neutrophil and mediate infection containment (101). NETs also play a role in thrombus formation. Evidence indicates that, in patients with COVID-19 infection, there is an upsurge of NET production that further propagates inflammation and thrombosis (101–103). The NET formation is characterized by elevated levels of cell-free DNA, myeloperoxidase-DNA (MPO-DNA), and citrullinated histone H3 (Cit-H3) (25, 101). A study of normal subjects compared with patients with COVID-19 infection has shown that the latter have higher levels of cell-free DNA, MPO-DNA, and Cit-H3 with both MPO-DNA and Cit-H3 being significantly elevated in mechanically ventilated patients (101). Pulmonary autopsies of patients with confirmed COVID-19 patients also confirm the presence of NET-containing microthrombi with neutrophil–platelet infiltration (103, 104).

The evidence of the role of NETs in AIS is well-described (17, 18, 105, 106, 114). Laridan et al. examined specimens of thrombi of patients undergoing endovascular thrombectomy and found that Cit-H3, the hallmark for NETs, was observed in the majority of the samples (17). Interestingly, patients with cardioembolic etiology have been shown to have a higher burden of NETs (102). Another study revealed that the presence of NETs may convey reperfusion resistance to mechanical and systemic revascularization procedures (17). Whether the role of neutrophils and NET in coronavirus-related stroke is secondary to micro- or macrothrombosis needs further elucidation.

### Viral-Induced Coagulation Dysfunction

Another important mechanism resulting in life-threatening systemic thromboembolic events among patients with coronavirus infection is the disruption of the coagulation pathways. Pro- and hypercoagulation affecting macro- and microvascular systems have also been implicated in various vascular events such as ischemic strokes. The International Society of Thrombosis and Hemostasis (ISTH) recognizes this unique phenomenon of sepsis-induced coagulopathy and proposed monitoring of platelet, D-dimer, PT, and fibrinogen among COVID-19 patients needing admission (115).

#### Fibrinolytic Shutdown

The imbalance between coagulation and fibrinolysis favoring the former manifests hematologically as an increase in D-dimer levels (115, 116). This is an immunological defense for the body to contain the viral infection, which results in thrombi formation (116). Furthermore, this implies a shutdown in the fibrinolytic system to clear the necrotic debris leading to massive fibrin formation, which also orchestrates coagulopathy (117). This shutdown has been further analyzed among critically ill COVID-19 patients where it has been demonstrated that, apart from the elevation D-dimer levels, more than 50% of the patients failed to lyse clots on thromboelastography (118–120) and hence increasing the likelihood of AIS as a further manifestation of COVID-19.

The tendency for 5% (this is still a gross underestimation, as we may never know the exact number strokes among deaths and even the mild strokes that might not present to the hospitals, as almost 40% of the stroke patients are not attending the hospitals during the pandemic time) of patients with COVID-19-related strokes to show spectacularly elevated D-dimer levels has been reported in several studies (91, 121, 122). Among the 32 cases reported by Yaghi et al., almost 50% showed D-dimer levels elevated to more than five times the normal range (94). Five of the six cases described by Beyrouti et al. also demonstrated D-dimer levels elevated more than 5–150 times above the normal limit (72). Avula et al., Lodigiani et al., Oxley et al., Berekashvili et al., and Wijeratne et al. also observed similar trends in the patients they described (3, 85, 88, 91, 123).

Venous thrombosis has also been described in a number of cases of COVID-19 infection resulting in fatal outcomes (107). Unsurprisingly, D-dimer levels were disproportionately elevated among patients with cerebral venous thrombosis (107, 108, 124). Another biomarker of fibrinolytic shutdown is the accumulation of fibrinogen and fibrin degradation products (119), and again, such retrospective analysis comparing COVID-19 and non-COVID-19 patients revealed higher fibrinogen levels in the former (123). Similarly, patients described in the literature with stroke and active COVID-19 infection have elevated serum fibrinogen levels (3, 72, 85).

The abnormalities of the coagulation biomarkers associated with COVID infection have been used to justify the use of systemic anticoagulation even in the acute stroke period. However, the outcomes for COVID patients are inconsistent (89, 123). Two of the five patients described by Lodigiani et al. who received Nadroparin died, and two of the four patients in Tunc's case series remain bedridden despite heparinization at the time of the publication (89, 123). Among the 32 COVID-19-related AIS described by Yaghi et al., 25 of whom received anticoagulation, more than 80% died or remained critically ill at the time of the publication (94). While the reason for poor neurological outcomes among these patients is multifactorial, a potential contributor is heparin resistance, especially among critically ill COVID-19 patients (24, 125).

For the small number of cases of COVID-19 patients who have also suffered AIS and received reperfusion therapy, consisting of intravenous thrombolysis or endovascular thrombectomy or a combination of both, the outcomes have not been particularly positive. In a case series regarding four patients with COVID-19-related AIS who also received systemic thrombolysis, all died (126). Similarly, in COVID-19-infected stroke patients reported in a healthcare system in New York who received alteplase and mechanical thrombectomy, the neurological outcomes were also poor (94). Currently, there is inadequate data to determine whether patients with COVID-19-related strokes may have resistance to tPA; however, it is known that the activation of the renin–angiotensin–aldosterone system results in the formation of plasminogen activator inhibitor (PAI-1), an inhibitor of tPA (127). Thus, it remains to be investigated whether these poor outcomes are related to counteractive mechanisms of PAI-I, increased thrombus burden, or resistance to tPA (128).

#### The Role of Natural Anticoagulants and Antiphospholipid Antibodies

Hypercoagulability is another sequela of COVID-19, especially in critically ill patients. One of the putative mechanisms, to which this is attributed, is the depletion of physiological anticoagulants and increased levels of coagulant factors and antiphospholipid antibodies (129–131). In a cross-sectional study of COVID-19-positive critically ill patients in China, it has been shown that the plasma levels of natural anticoagulants such as protein C, protein S, and antithrombin were below physiologically normal levels (127). The same study revealed that more than 50% of the patients developed antiphospholipid antibodies (127) that have been suggested to play a role in the pathogenesis of COVID-19-related strokes. Zhang et al. described three patients with multiple cerebral infarctions who tested positive for anticardiolipin immunoglobulin A (IgA) antibodies as well as anti-β_2_-glycoprotein I IgA and IgG antibodies (130). On the other hand, five of the six COVID-19-associated stroke patients described by Beyroutti et al. did not show antiphospholipid antibodies, but all of them were positive for lupus anticoagulant (LA) (72). Two other severe stroke patients with poor neurological outcome and concomitant COVID-19 infection have also been reported as testing negative for both antiphospholipid antibodies and LA (99, 100). Thus, while there is inconsistency in the trend of these procoagulant factors in ischemic stroke patients, Zhang has proposed a possible secondary antiphospholipid antibody syndrome especially in patients who have thrombotic manifestations and positive serum antibodies (130). If this was the case, then the initiation of therapeutic anticoagulation would be required for these patients (130). However, it has not translated to screening of all COVID-19 patients, as a transient increase in these parameters is expected in viral infections (129).

### The Role of the Endothelium

A component of Virchow's triad, endothelial dysfunction is another major contributor to SARS-CoV-associated thrombosis. The virus has been shown to demonstrate a predilection to invade vascular endothelial cells by attaching to the ACE2 protein, which facilitates subsequent invasion (9, 132, 133).

#### Endothelium-Driven Activation of the Extrinsic Coagulation System

COVID-19 promotes an imbalance between ACE2 and angiotensin II (AT-II) receptors leading to the upregulation of the latter, which is responsible for the tonic production of platelet tissue factor (TF or factor111) in platelets and macrophages, and further interaction with factor VII to activate the extrinsic coagulation system (134, 135). TF is essential for fibrin formation at the site of vascular injury and may also be an important factor contributing to thrombogenesis, especially with the induction of inflammatory cells originating from the subendothelium (26).

Increased expression of TF has previously also been shown to be associated with thrombus formation in patients with ischemic stroke (109, 110). The EPICOR study in 2015 revealed that patients with elevated TF doubled the risk of stroke (109). Experimental studies also demonstrated that the inhibition of TNF-α-induced TF significantly reduces atheromatous plaque formation (30). Whether inflammation-induced tissue factor formation has a role in intracranial thrombosis in COVID patients particularly remains speculative.

#### Endothelium-Driven Nitrous Oxide Deficiency

Healthy endothelium is necessary to produce nitric oxide (NO), which is known as a vasodilator and plays a role in preventing thrombosis (27). SARS-CoV2-related endotheliopathy results in the suppression of nitric oxide synthase (NOS), resulting in nitric oxide deficiency (133). *In vitro* studies performed in 2009 on SARS-CoV also revealed that nitrous oxide inhibited viral entry replication by decreasing spike (S) protein expression and via its effects on the cysteine proteases encoded in the SARS-CoV (133).

Endothelium-derived nitrous oxide affects the cerebral circulation by its tonic vasodilatory effect and the prevention of adhesion of platelets and leukocytes to the vessel wall, both of which have an obligatory role in stroke pathogenesis (28). A study comparing stroke patients and healthy controls revealed that the levels of asymmetrical dimethylarginine (ADMA), a NOS inhibitor, is increased in the former, making it a risk factor for stroke (29). To date, few studies, if any, are looking into the potential role of NO in the treatment and prevention of COVID-19-related strokes; however, there is evidence to suggest that NO may also have a potential benefit for pulmonary-related COVID-19 complications (136).

## The Role of the Renin–Angiotensin–Aldosterone System and Ace2 Deficiency

A unique characteristic of the SARS-CoV2 infection is its avid interaction with the ACE2 receptors, which affect the RAAS to a significant degree (137). It is known that viral-induced ACE2 deficiency results in significant alterations in the balance between the classical RAAS and the counterregulatory ACE2, which is essential for maintaining homeostatic mechanisms in the body (137). In particular, ACE2, which is ubiquitous in the brain, heart, and the vascular systems protects patients from the organ-damaging effects of the classical RAAS (138). This COVID-19-related disruption of the RAAS axes is likely to contribute to stroke pathogenesis, as it promotes inflammation, vasoconstriction, and end-organ damage (139, 140).

Various COVID-19 registries have shown that there is an increased fatality rate among patients with preexisting cardiovascular diseases such as hypertension and diabetes (140). Similarly, a majority of patients with AIS and COVID-19 report hypertension as a classical risk factor (111). The contribution of hypertension in the outcomes of patients with COVID-19 infection is likely explained in various mechanisms. Still, overactivity of the classical RAAS along with viral-induced endothelial vasoconstriction may be factors adding to the neurological exacerbation of COVID infection (140). Worse neurological outcomes among patients with COVID and ischemic strokes may be attributable to the unimpeded activity of the classical RAAS pathway in the brain, which has also been implicated in higher brain functions such as memory and cognition (141). Furthermore, upregulation of the classical RAAS pathway is likely to result in the overactivity of the sympathetic nervous system, which may exacerbate preexisting traditional risk factors for stroke such as hypertension, diabetes, and cardiac dysrhythmias (142).

## Covid, Neurological Manifestations and Implications for Neurorehabilitation

Neurorehabilitation for patients recovering from severe COVID and AIS is set to be extra challenging in times of pandemic with its requirements for social distancing and, in many cases, need for online delivery in the immediate post-COVID stages. Currently, there are little reliable data about how well individuals with severe respiratory system and lung damage will recover or whether scarring will make such patients vulnerable to other viral infections (31). Furthermore, questions remain regarding whether the hypoxia engendered during the acute hospitalization phase will have lasting effects on the brain and nerve tissue especially on high metabolic demand areas including the sympathetic system and the fast visual attention and eye movements pathways (112) and long motor pathways.

Helms et al. first described the occurrence of dysexecutive syndrome in 36% of patients of critically ill COVID survivors in a French cohort characterized by lack of attention and disorganized movement with commands (143). Various case reports likewise described cases of patients who had severe impairments in executive function in the postacute stage who had significant recovery after aggressive neurorehabilitation (37, 144). In the same manner, varying degrees of depression and anxiety have also been identified after the acute stage of infection among COVID survivors (36, 145, 146). Interestingly, a meta-analysis of observational studies also notes that, among patients with previous SARS and MERS-CoV infection, neuropsychiatric manifestations such as depression, anxiety, irritability, memory impairment, sleep deprivation, and posttraumatic disorder were strikingly prevalent (147). One would also expect that psychological anxiety and the physiologically high immune responses (psychoneuroimmunological outcomes) would also lead to various psychiatric sequelae that have adverse effects on mental health of patients (32, 148). The neurological sequelae of SARS-CoV2 infection have gained recent publicity in social and print media (Facebook “Long haul COVID support groups”). Symptoms include persistent “brain fog,” fatigue, breathlessness, anxiety, depressive mood, and motor weakness, affecting subsets of young, middle aged, and older patients including a significant proportion who showed only mild symptoms, during the infected stage (40, 149). We have suggested that these symptoms comprise aspects of *PCNS* and hypothesized that such a syndrome is due to persistent inflammation following COVID-19 infection (113, 150). These complications are undeniably posing significant challenges ahead for the neurorehabilitation fraternity.

Mathew et al. identified a subgroup of patients with T-cell activation characteristic of acute viral response plasmablast responses reaching over 30% of the circulating cells with three different immunotypes associated with poor clinical trajectories supporting the current understanding of the pathobiology (151).

Fridman et al. found the frequency of stroke to be high among patients with severe COVID-19 with a very high inpatient mortality across all age groups demonstrating further evidence to the shared pathobiology as one go through the cluster analysis of this paper (143).

Overall, our review of the pathobiological basis of COVID-19 demonstrates that it is a multisystem illness with high likelihood of long-term physical, cognitive, psychological, and social sequalae in those who survive the illness. The scale of the burden of the disease and impact is enormous globally.

Furthermore, there is also no doubt that the current pandemic is overwhelming the neurorehabilitation sector globally. While it is too early to comment about the potential pandemic of long-term neurological issues for the millions of infected patients worldwide, the current review predicts the high likelihood of this possibility. Similarly, Boldrini et al. has noted that the two main factors already impacting the neurorehabilitation sector during the first phase of the pandemic (144) are the following:

(i) increased pressure from the acute care services to transfer the patients to rehabilitation medicine services;
(ii) increased difficulties without patient rehabilitation activities and home-based rehabilitation due to the restrictions enforced by the local and national authorities (144).

Indeed, the challenges described by Boldrini et al. above make the situation even more complex. It is critical to create an efficient healthcare delivery system tailored to each individual, each community, and each country. It is equally important to understand the biological underpinning behind the illness and post-COVID-19 complications. Tailor-made, individualized, targeted neurorehabilitation program should be developed with the available resources (e.g., telemedicine can be tested with easily available smart phone-based deliverables such as WhatsApp, Viber, Facetime, and Google Talk where professional personnel are not always available and sophisticated technology are not around). Family members can be a really useful resource in these challenging circumstances (36, 37, 145, 146).

To this end, we recommend considerations of Mantovani et al. (152) who recently collected and reviewed relevant evidence-based recommendations on the efficacy of cognitive rehabilitation, based on a comprehensive evaluation of 491 papers presenting the latest evidence-based practice for neurorehabiliation of traumatic brain injury (TBI) patients whose pathobiology bears many similarities to that presented in this review in the context of COVID-19 and systemic involvement (147, 152, 153).

Mantovani et al. as well offer a road map and a comprehensive perspective on the role of tele-neurorehabilitation and virtual rehabilitation to gain adequate cognitive stimulation in the era of physical distancing and lockdowns related to COVID-19 pandemic, reminding us of the potential opportunities globally (152). For further details, see Mantovani et al. and the 29 recommendations on evidence-based cognitive rehabilitation in the context of stroke and TBI nominated by the Cognitive Rehabilitation Task Force (CRTF), which suggests that a more comprehensive biological understanding of the various systems affected by TBI should lead to more collaborative translational research utilizing artificial intelligence and novel technology to find better individually designed precision solutions (e.g., video-consults, tablet-based measurements, and use of wearable devices). Lastly, the integration of services, including private and public partnerships such as university–public hospital partnerships and partnerships with community organizations and volunteers, are likely to create excellent opportunities for the future (112, 154, 155) and better evidence-based neurorehabilitation pathways.

In support of this more generalized theoretical approach, our group has demonstrated here the expected neurological impact of shared pathobiology between AIS and COVID-19 here, and the debilitating condition that characterizes PCNS (150) manifests as chronic fatigue, impaired thinking, depression, anxiety, breathing difficulties, and muscle weakness (40, 40, 149, 150). Thus, we suggest that treatment with known Food and Drug Administration (FDA)-approved immunomodulatory hormones such as melatonin (14, 15, 33, 156–162) and nutraceuticals such as curcumin (163) might be expected to enhance the likelihood of PCNS patients benefitting from any neurorehabilitation interventions as a matter of priority.

## Conclusion

Our review has shown here that severe COVID-19 infection has the potential to lead to the disruption of most physiological systems and results in various multisystemic thrombotic phenomena including acute ischemic stroke. While inflammation orchestrates its pathogenesis, the further perturbation of the coagulation system resulting in fibrinolytic shutdown likewise contributes to neurological manifestation. Furthermore, the predilection of the virus to attach to ACE2 receptors in various cells, including the vascular endothelial system, may also disrupt the renin–angiotensin system, which further contributes to stroke pathogenesis. Clearly, there is a gap in the understanding of this phenomenon, and large-scale human and animal studies are necessary, as this co-occurrence results in deleterious outcomes.

The impact of COVID-19 in the human brain is as such that millions of patients are likely to experience problems with normal functioning requiring far more psychoneuroimmunological evidence-based understanding of neurorehabilitation services that are able to overcome incipient recovery problems.

## Data Availability Statement

The original contributions generated in the study are included in the article/supplementary material, further inquiries can be directed to the corresponding author.

## Author Contributions

TW conceived the idea. TW and CS performed the literature search independently. TW, CS, SC, and LK contributed to the manuscript writing with critical revisions. All authors read the final manuscript and agreed.

## Conflict of Interest

The authors declare that the research was conducted in the absence of any commercial or financial relationships that could be construed as a potential conflict of interest.
